# Multi-Mode Electromagnetic Ultrasonic Lamb Wave Tomography Imaging for Variable-Depth Defects in Metal Plates

**DOI:** 10.3390/s16050628

**Published:** 2016-05-02

**Authors:** Songling Huang, Yu Zhang, Shen Wang, Wei Zhao

**Affiliations:** State Key Lab of Power Systems, Department of Electrical Engineering, Tsinghua University, Beijing 100084, China; zhang-yu13@mails.tsinghua.edu.cn (Y.Z.); wangshen@mail.tsinghua.edu.cn (S.W.); zhaowei@mail.tsinghua.edu.cn (W.Z.)

**Keywords:** multi-mode Lamb waves (LWs), variable-depth defects, electromagnetic acoustic transducers (EMATs), cross-hole tomography imaging (CMI)

## Abstract

This paper proposes a new cross-hole tomography imaging (CTI) method for variable-depth defects in metal plates based on multi-mode electromagnetic ultrasonic Lamb waves (LWs). The dispersion characteristics determine that different modes of LWs are sensitive to different thicknesses of metal plates. In this work, the sensitivities to thickness variation of A0- and S0-mode LWs are theoretically studied. The principles and procedures for the cooperation of A0- and S0-mode LW CTI are proposed. Moreover, the experimental LW imaging system on an aluminum plate with a variable-depth defect is set up, based on A0- and S0-mode EMAT (electromagnetic acoustic transducer) arrays. For comparison, the traditional single-mode LW CTI method is used in the same experimental platform. The imaging results show that the computed thickness distribution by the proposed multi-mode method more accurately reflects the actual thickness variation of the defect, while neither the S0 nor the A0 single-mode method was able to distinguish thickness variation in the defect region. Moreover, the quantification of the defect’s thickness variation is more accurate with the multi-mode method. Therefore, theoretical and practical results prove that the variable-depth defect in metal plates can be successfully quantified and visualized by the proposed multi-mode electromagnetic ultrasonic LW CTI method.

## 1. Introduction

The dispersion and multi-mode characteristics of ultrasonic Lamb waves (LW) indicate that the group velocity of a particular mode of LW varies with the product of frequency and waveguide thickness (frequency–thickness product, FTP) [1,2]. Based on the dispersion characteristics, the cross-hole tomography imaging (CTI) method utilizes the time-of-flight (TOF) to reconstruct the thickness distribution of the defect area so as to obtain the image of the defect [3,4,5]. The concerned imaging area is divided into M × N meshes, and the transmitters and receivers are respectively arranged along the two sides of the imaging area. When some LW rays pass through the meshes with defects, the group velocities will change with the area thickness, and this leads to the changes in the TOF of the LW rays. The distribution of the slowness (the reciprocal of the group velocity) can be calculated by solving:

(1) $$T_{i}=\sum_{j=1}^{n}L_{ij}*S_{j}\;(i=1,2,...,m)$$

where *L_ij_* is the length of the *i-*th LW ray in the *j-*th mesh, *T_i_* is the TOF of the *i-*th LW ray, *S_j_* is the slowness of the *j-*th mesh, *m* is the total number of LW rays, and *n* is the total number of meshes.

Each particular mode of LW has different through-thickness displacement values, and each is sensitive to different depths of defects [6]. The majority of CTI research is focused on image reconstruction for fixed-depth defects, but the imaging for variable-depth defects suffers relatively poor quality, and the depth variation can hardly be reconstructed. The relationship between detecting signals and the depths of defects has been studied [7,8,9,10], but the research objects had different defects of different depths, as opposed to varying depths within a single defect. Endoh demonstrated a photoacoustic microscope (PAM) to detect metal planes with titled or variable-depth subsurface defects [11], while the detecting accuracy was limited because the image of the defect was not constructed.

Related research about multi-mode LW CTI is mostly confined to comparing the imaging quality of different experimental methods using different modes of LWs, and only one particular mode of LW is applied in each imaging experiment. A continuous wavelet transform method was used in Ho’s work to analyze multi-mode LW propagation [12]. However, at present, there is nearly no practical multi-mode LW CTI method for variable-depth defects.

This work attempts to define the sensitivity of multi-mode LWs in terms of working frequency and waveguide thickness, to explore the variation pattern of the defined sensitivity, and to propose a novel multi-mode electromagnetic LW CTI method for variable-depth defects in metal plates.

## 2. LW’s Sensitivity to Thickness

**Figure 1:**
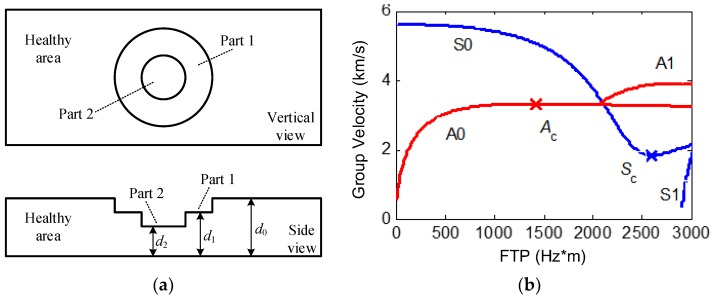
Research object in an aluminium plate. (**a**) A typical circular defect with variable depth and (**b**) the dispersion curves of a Lamb wave (LW)’s group velocity in the aluminium plate.

To be clear, in the near and local range of a given defect, the thickness of the area outside the defect is defined as the “fundamental thickness” in this work. Figure 1a illustrates a typical circular defect with variable depth in an aluminium plate. According to the above definition, the fundamental thickness of Part 1 is *d*_0_, and the fundamental thickness of Part 2 is *d*_1_.

Based on the dispersion characteristics of LWs, higher-order LWs can hardly propagate through the defect area where the FTP is relatively small. Therefore, only A0- and S0-mode LWs are applied in the CTI in this work, and the working points are selected at the relatively small FTP area in order to avoid the interference of multi-mode LWs.

According to the dispersion equations of LWs [13], the dispersion curves of a LW’s group velocity in the small FTP area (less than 3000 Hz*m) of an aluminium plate are calculated and shown in Figure 1b, where the longitudinal wave velocity and transverse wave velocity are respectively evaluated as 6300 m/s and 3300 m/s. The working point A_c_ is the demarcation of an A0-mode LW’s group velocity monotonicity varying with the FTP. The group velocities of working points on the left side of A_c_ will get bigger as the FTP grows, and those on the right side of A_c_ will get smaller as the FTP grows. Therefore, in this work, the working point A_c_ is defined as the cut-off working point of an A0-mode LW, and the corresponding FTP *x*_a_ is defined as the cut-off FTP of an A0-mode LW. In a similar way, the working point S_c_ in Figure 1b is defined as the cut-off working point of an S0-mode LW, and the corresponding FTP *x*_s_ is defined as the cut-off FTP of an S0-mode LW.

In this work, the LW’s sensitivity to thickness *S*_sen_(*f*, *d*) at a given working point is defined as the rate of change of a LW’s theoretical slowness of thickness variation. Therefore, the sensitivity of a particular mode of LW *S*_sen_(*f*, *d*) can be expressed as the absolute partial derivative of a LW’s theoretical slowness *S_j_* of its fundamental thickness *d*:

(2) $$S_{sen}(f,d)=\left|\frac{\partial S_{j\_the}(f,d)}{\partial d}\right|$$

where *S_j_*__the_(*f*, *d*) is the theoretical slowness of a particular mode of LW. Since the sensitivity of each mode of LW will vary in fundamental thickness, relatively high sensitivity at a given fundamental thickness can hardly be guaranteed if only one single-mode LW is applied in the CTI. Therefore, by the complementation of a multi-mode LW, there will almost always be at least one mode of LW that is relatively highly sensitive to different fundamental thicknesses so as to greatly improve the imaging quality of a variable-depth defect by the multi-mode LW CTI method.

**Figure 2:**
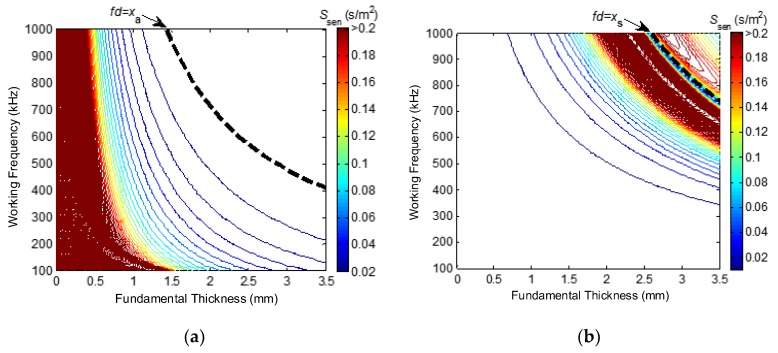
Two-dimensional distribution of sensitivity *S*_sen_(*f*, *d*) on the frequency–thickness plane. (**a**) A0-mode LW and (**b**) S0-mode LW.

On the basis of dispersion curves in Figure 1b, the definition of slowness in Equation (1) and the definition of a LW’s sensitivity in Equation (2), the variation curves of A0- and S0-mode LW sensitivity *S*_sen_(*f*, *d*) varying in fundamental thickness at different working frequencies can be respectively calculated and illustrated as the two-dimensional distribution in the frequency–thickness plane, as shown in Figure 2. In consideration of a LW’s high attenuation due to a higher frequency, as well as its poor recognition of defects due to a lower frequency, the working frequencies in this work are selected as 100 kHz to 1000 kHz. Different values of sensitivity *S*_sen_(*f*, *d*) are expressed as different contour lines in Figure 2, and the difference values between adjacent contour lines are the same.

As illustrated in Figure 2a, the sensitivity of an A0-mode LW decreases as the fundamental thickness or working frequency increases. The dotted line represents the FTP that is equal to the cut-off FTP of the A0-mode LW *x*_a_, and the working area should be at the lower left part of the dotted line. For a given working frequency, the sensitivity is relatively high when the fundamental thickness is relatively small. Figure 2b illustrates that the sensitivity of an S0-mode LW increases as the fundamental thickness or working frequency increases and when the FTP is less than the cut-off FTP of an S0-mode LW. The dotted line represents the FTP that is equal to the cut-off FTP of an S0-mode LW *x*_s_, and the working area should be at the lower left part of the dotted line. For a given working frequency, the sensitivity is relatively high when the fundamental thickness is relatively large.

The sensitivity of an S0-mode LW decreases near the point S_c_ (cut-off FTP) because the derivative of group velocity curve almost reaches zero. Furthermore, the cut-off curve *fd* = *x*_s_ is defined according to the cut-off FTP. In theoretical research, the cut-off FTP can be decreased to less than the FTP of point S_c_ so as to prevent the working area of the S0-mode LW from falling into the near neighbor of S_c_. In practical detection and imaging for defects, the working area of an S0-mode LW nearly never reaches close to the near neighbor of S_c_, because the thickness of the defect area is always smaller than the thickness of the healthy area. Therefore, the potential poor sensitivity of an S0-mode LW near the neighbor of S_c_ can be theoretically and practically avoided.

The sensitivity value of a corresponding selected contour line is defined as the sensitivity threshold *S*_TH_ in this work, for both A0-mode and S0-mode LWs. Further, the area in which *S*_sen_(*f*, *d*) is greater than *S*_TH_ is defined as the sensitive area. Therefore, the A0-mode LW is more sensitive to thickness variation when the thickness is relatively small, and the S0-mode LW is more sensitive when the thickness is relatively large. This lays the theoretical foundation for the cooperation principle and method of multi-mode LW CTI for variable-depth defects.

## 3. Principles and Procedures of Multi-Mode LW CTI

On the basis of the A0-mode and S0-mode LWs’ sensitivity to FTP, the cooperation principles for multi-mode LW CTI are provided as:

(1) The FTP of an A0-mode LW and an S0-mode LW should be respectively less than *x*_a_ and *x*_s_, which are respectively the cut-off FTP of an A0-mode LW and an S0-mode LW, in order to preserve the monotonicity of the LW group velocity.
(2) Based on the sensitivity threshold *S*_TH_, the working areas of an A0-mode LW and S0-mode LW should be respectively located in their own sensitive areas to increase the imaging sensitivity.
(3) An A0-mode LW should be mainly applied in a small-thickness situation, and an S0-mode LW should be mainly applied in a large-thickness situation, according to the investigation results of LW’s sensitivity to thickness.
(4) The working areas of an A0-mode LW and an S0-mode LW at their own working frequencies should partially overlap each other in order to guarantee that, for a given thickness, at least one mode of LW is sensitive. Specifically, the upper limit thickness of an A0-mode LW working area should be greater than the lower limit thickness of an S0-mode LW working area.

**Figure 3:**
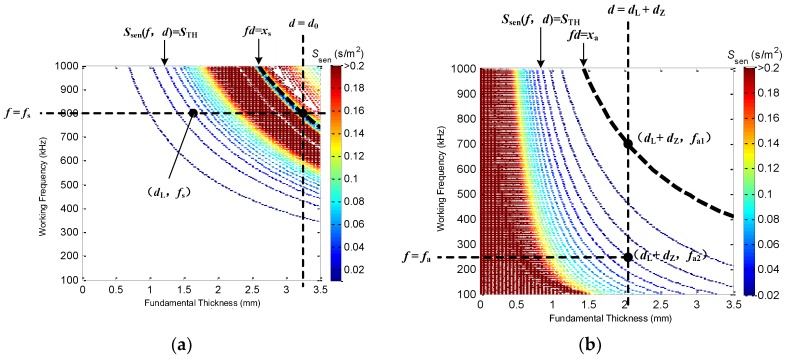
Principles for determining the working parameters. (**a**) S0-mode LW working parameters and (**b**) A0-mode LW working parameters.

The determination of working parameters and operating points of an A0-mode LW and S0-mode LW is significantly important for their cooperation and thus multi-mode LW CTI. Figure 3 illustrates the principles and methods for determining the working parameters of an A0-mode LW and an S0-mode LW.

The procedures for working parameter and operating point determination are as follows:

(1) Set the sensitivity thresholds *S*_TH_ respectively for the A0-mode and S0-mode LWs.
(2) Locate the curve *fd* = *x*_s_ in Figure 3a, which is the sensitivity distribution of the S0-mode LW. The working area of the S0-mode LW should be at the lower left part of the curve.
(3) Locate the straight line *d* = *d*_0_ in Figure 3a, where *d*_0_ is the thickness of the healthy area. The working area of the S0-mode LW should be confined to the left part of the straight line. Because the S0-mode LW is more sensitive when the thickness is relatively large, the thickness of the healthy area (known quantity) should be figured on the sensitivity distribution plane of the S0-mode LW, and the working areas of the S0-mode LW should firstly be determined.
(4) Calculate the intersection point coordinates (*d*_0_, *f*_s_) of the curve *fd* = *x*_s_ and the straight line *d* = *d*_0_, and take *f*_s_ as the working frequency of the S0-mode LW. If the working frequency of the S0-mode LW is bigger than *f*_s_, then the working area will not cover the thickness of the healthy area, and, if the working frequency is smaller than *f*_s_, the sensitive thickness range that the S0-mode LW covers greatly decreases. Therefore, *f*_s_ at the intersection point is almost the perfect working frequency of the S0-mode LW.
(5) Locate the contour line *S*_sen_(*f*, *d*) = *S*_TH_ in Figure 3a, and the working area of the S0-mode LW should be confined to the upper right part of the contour line, which is the sensitive area of the the S0-mode LW.
(6) Calculate the intersection point coordinates (*d*_L_, *f*_s_) of the straight line *f* = *f*_s_ and the contour line *S*_sen_(*f*, *d*) = *S*_TH_ to obtain the lower limit *d*_L_, and the working area of the thickness of the S0-mode LW at working frequency *f*_s_ is [*d*_L_, *d*_0_]. The sensitive-thickness range of the S0-mode LW is maximized by the method proposed above.
(7) Set the overlapping width *d*_Z_(*d*_Z_ > 0) of the A0-mode and S0-mode LWs working areas in Figure 3b to obtain the working area of the thickness of the A0-mode LW [0, *d*_L_ + *d*_Z_]. The overlapping width is to ensure that the sensitive working thickness by the cooperation of the A0-mode and S0-mode LWs can completely cover the thickness of the variable-depth defect area.
(8) Locate the curve *fd* = *x*_a_ in Figure 3b. The working area of the A0-mode LW should be confined to the lower left part of the curve, which is the cut-off FTP of the A0-mode LW
(9) Calculate the intersection point coordinates (*d*_L_ *+ d*_Z_, *f*_a1_) of the curve *fd* = *x*_a_ and the straight line *d* = *d*_L_ *+ d*_Z_.
(10) Locate the contour line *S*_sen_(*f*, *d*) = *S*_TH_ in Figure 3b, and the working area of the A0-mode LW should be confined to the lower left part of the contour line, which is the sensitive area of the the A0-mode LW.
(11) Calculate the intersection point coordinates (*d*_L_ *+ d*_Z_, *f*_a2_) of the contour line *S*_sen_(*f*, *d*) = *S*_TH_ and the straight line *d* = *d*_L_ *+ d*_Z_.
(12) Select the smallest of *f*_a1_ and *f*_a2_ as the working frequency *f*_a_ of the A0-mode LW. If the contour line *S*_sen_(*f*, *d*) = *S*_TH_ is under the curve *fd* = *x*_a_, *f*_a2_ is selected as the working frequency *f*_a_. Otherwise, if the contour line *S*_sen_(*f*, *d*) = *S*_TH_ is above the curve *fd* = *x*_a_, *f*_a1_ is selected as the working frequency *f*_a_. This is to maximize the sensitive working thickness of the A0-mode LW to be [0, *d*_L_ + *d*_Z_].

After the determination of working parameters and operating points of the A0-mode LW and S0-mode LW, the procedures of multi-mode LW CTI can be concluded:

(1) Calculate the working frequencies and working areas of the A0-mode and S0-mode LWs based on the proposed cooperation principles.
(2) Respectively implement the single A0-mode and single S0-mode LWs to detect the defect area and obtain the TOF.
(3) Conduct the CTI respectively based on the A0-mode and S0-mode LWs using the TOF.
(4) Conduct data fusion of CTI results from the A0-mode and S0-mode LWs.

The CTI results of the single A0-mode and single S0-mode LWs are respectively obtained based on the above working parameters, and the data fusion of CTI results from the A0-mode and S0-mode LWs is to be conducted. For a certain imaging mesh, its thickness, calculated from single A0-mode LW CTI, is displayed as *d*_a_, and its thickness, calculated from single S0-mode LW CTI, is displayed as *d*_s_. According to the working parameters and operating points of the A0-mode and S0-mode LWs, the sensitive thickness range of the A0-mode LW is [0, *d*_L_ + *d*_Z_], and the sensitive thickness range of the S0-mode LW is [*d*_L_, *d*_0_].

(1) In the condition of *d*_a_∈[0, *d*_L_ + *d*_Z_] and *d*_s_∈[*d*_L_, *d*_0_], or in the condition of *d*_a_∉[0, *d*_L_ + *d*_Z_] and *d*_s_∉[*d*_L_, *d*_0_], the mesh thickness after fusion is *d*_add_ = 0.5*d*_a_ + 0.5*d*_s_. In this situation, *d*_a_ is in the sensitive thickness range of the A0-mode LW, and *d*_s_ is in the sensitive thickness range of the S0-mode LW (or neither *d*_a_ nor *d*_s_ is in the corresponding sensitive thickness range), which means the calculated thicknesses are both expected ones, so the weighted average value is selected as the final thickness of the mesh.
(2) In the condition of *d*_a_∈[0, *d*_L_ + *d*_Z_] and *d*_s_∉[*d*_L_, *d*_0_], the mesh thickness after fusion is *d*_add_ = *d*_a_. In this situation, only *d*_a_ is in the sensitive thickness range of the A0-mode LW, but *d*_s_ is not in the sensitive thickness range of the S0-mode LW, so the calculated thickness *d*_s_ should not be considered. That means the mesh thickness is in the sensitive thickness range of the A0-mode LW [0, *d*_L_ + *d*_Z_]; therefore, *d*_a_ is selected as the final thickness of the mesh.
(3) In the condition of *d*_a_∉[0, *d*_L_ + *d*_Z_] and *d*_s_∈[*d*_L_, *d*_0_], the mesh thickness after fusion is *d*_add_ = *d*_s_. In this situation, only *d_s_* is in the sensitive thickness range of the S0-mode LW, but *d_a_* is not in the sensitive thickness range of the A0-mode LW, so the calculated thickness *d_a_* should not be considered. That means the mesh thickness is in the sensitive thickness range of the S0-mode LW [*d*_L_, *d*_0_]; therefore, *d_s_* is selected as the final thickness of the mesh.

## 4. Experiments and Results

**Figure 4:**
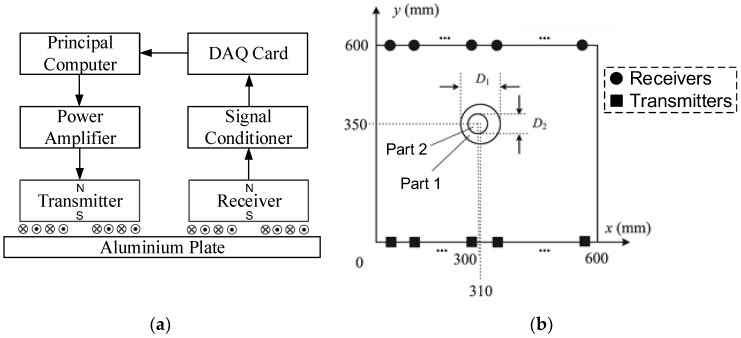
Experimental platform for multi-mode LW cross-hole tomography imaging (CTI). (**a**) Schematic diagram of the experimental system and (**b**) location parameters of the experiment.

The multi-mode LW CTI method was implemented on a 3-mm thick aluminum plate with a variable-depth defect. Figure 4a illustrates the schematic diagram of the experimental system. The contra-flexure coils (CFC) were applied as the transmitting and receiving coils for the transmitters and receivers to separately stimulate and receive near-pure single A0-mode and S0-mode LWs [14]. The pure single-mode EMAT was realized by the configuration of the distance *l* between the two adjacent annular wires along the radial direction, which is supposed to be half of the wavelength of the desired mode of LW. The transmitters were excited by the power amplifier, which was controlled by the principal computer. The LW were converted into electrical signals by the receivers. Then, the detected signals were amplified and filtered by the signal conditioner and subsequently collected by the DAQ card. The collected data were finally sent to the principal computer to be analyzed for LW CTI.

The 600 mm × 600 mm imaging area in the aluminum plate was divided into 120 × 120 meshes, as shown in Figure 4b. The variable-depth defect was composed of two parts: Part 1 is located at (310 mm, 350 mm), the diameter *D*_1_ is 120 mm, and the depth *d*_01_ is 1 mm; Part 2 is located at (300 mm, 350 mm), the diameter *D*_2_ is 60 mm, and the depth *d*_02_ is 2 mm. It can be seen that the fundamental thickness of Part 1 is 3 mm, and the fundamental thickness of Part 2 is 2 mm.

Based on the proposed principles and procedures of LW CTI, the calculated working frequencies of the A0-mode and S0-mode LWs were respectively 270 kHz and 700 kHz. Table 1 illustrates the structure parameters of the single A0-mode EMAT and the single S0-mode EMAT. For each single-mode LW detection, 14 transmitters and 14 receivers were respectively located at the two sides of the imaging area.

Firstly, one of the A0-mode transmitters stimulated the pure A0-mode LW in the aluminum plate, all 14 A0-mode receivers received the pure A0-mode LW, and the 14 TOF were extracted by the short-time Fourier transform (STFT) method. Similar procedures were conducted for all 14 A0-mode transmitters, so there were a total of 196 (14 × 14) groups of TOF extracted from the single A0-mode LW detection experiment. Secondly, the S0-mode transmitters and receivers were implemented in the detection experiment, and the procedures were similar. Fourteen S0-mode transmitters respectively stimulated the pure S0-mode LW in the aluminum plate, and the 14 S0-mode receivers received the pure S0-mode LW. Thus, there were also a total of 196 (14 × 14) groups of TOF extracted from the single S0-mode LW detection experiment.

**Figure 5:**
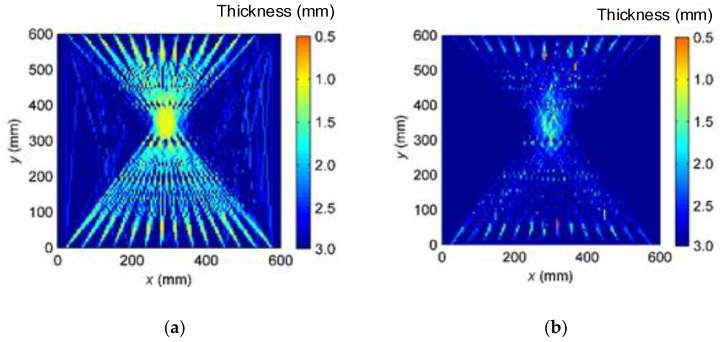

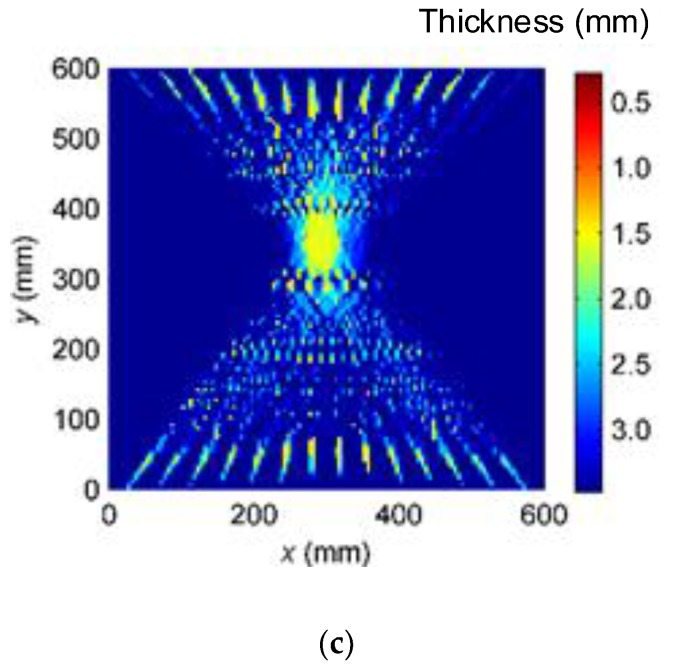
Thickness distribution results of LW CTI. (**a**) Single A0-mode LW CTI; (**b**) single S0-mode LW CTI; and (**c**) multi-mode LW CTI.

The 196 groups of TOF extracted from the A0-mode LW detection, and the 196 groups of TOF extracted from the S0-mode LW detection were respectively implemented in the simultaneous iterative reconstruction technique to reconstruct the defect image, in which the solution threshold was set as 0.05%, and the relaxation parameter was 0.1. The thickness distribution results of single A0-mode and single S0-mode LW CTI are respectively shown in Figure 5a,b.

As illustrated in Figure 5a, the reconstruction image and profile of Part 2 of the variable-depth defect are relatively clear when the single A0-mode LW is applied. However, many artifacts appear in the image, and the profile of Part 1 is nearly submerged by the artifacts. When the single S0-mode LW is applied, the reconstruction image and profile of Part 1 of the variable-depth defect are relatively clear. However, the image of Part 2 is seriously distorted, its profile severely blurred. Therefore, the reconstruction image and profile of the variable-depth defect suffer relatively low quality when only the single-mode LW is implemented in the CTI.

On the basis of the proposed data fusion principles and methods, the thickness distributions from the single A0-mode and single S0-mode LW CTI were fused, as illustrated in Figure 5c. The reconstruction images and profiles of both Part 1 and Part 2 of the variable-depth defect are relatively clear, and the artifacts are greatly reduced. Additionally, the fused thickness distribution from the multi-mode LW CTI is closer to the actual shape and profile of the variable-depth defect.

**Figure 6:**
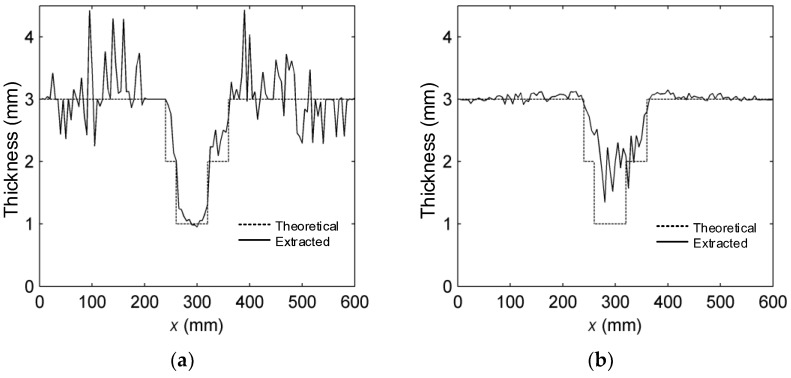

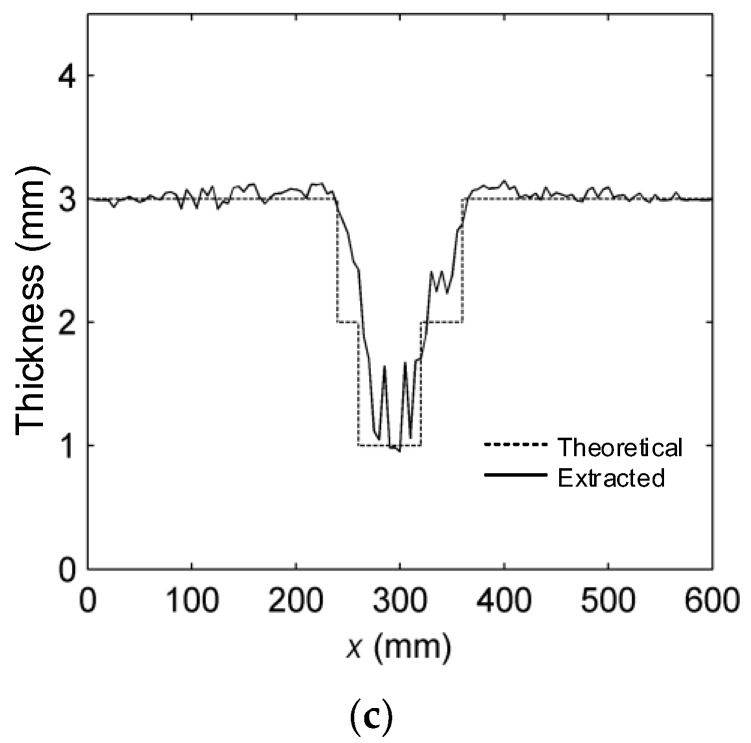
Thickness distribution curves at *y* = 350 mm from LW CTI. (**a**) Single A0-mode LW CTI; (**b**) single S0-mode LW CTI; and (**c**) multi-mode LW CTI.

The thickness distribution curves at *y* = 350 mm were respectively extracted from Figure 5a–c, and the results are respectively shown in Figure 6a–c.

It can be seen that, when the single A0-mode LW is applied, the thickness distribution curve of Part 2 fits the actual thickness curve quite well, but that of Part 1 suffers poor fitting. Figure 6b indicates that when the single S0-mode LW is applied, the thickness distribution curve of Part 1 fits the actual thickness curve well, but that of Part 2 can hardly reflect the actual thickness curve.

The thickness distribution curve extracted from the multi-mode LW CTI shows an obvious ladder shape, as illustrated in Figure 6c. The calculated thickness distribution curve displays a relatively higher degree of fitting for the actual thickness curves of both Part 1 and Part 2.

Therefore, the proposed multi-mode LW CTI method is proven to be able to accurately quantify and visualize variable-depth defects in metal plates.

## 5. Conclusions

This work proposes a novel multi-mode electromagnetic ultrasonic LW CTI method for variable-depth defects in metal plates. The dispersion characteristics of LWs indicate that different modes of LWs are sensitive to different thicknesses of metal plates, and the sensitivity of a given mode of LWs varies with the thickness. Moreover, the single-mode LW CTI method has here been shown to suffer low resolution when it is used in variable-depth situations. The sensitivities of A0-mode and S0-mode LWs on thickness variation were theoretically studied and demonstrated on a frequency–thickness plane. Related results show that, for certain working frequencies, A0-mode LW is more sensitive to thickness variation on small thickness areas, and S0-mode LW is more sensitive on large thickness areas. Based on the above theoretical results, the principles and procedures for the cooperation of A0- and S0-mode LW CTI are proposed. Additionally, the experimental platform for multi-mode LW CTI on an aluminum plate with a variable-depth defect was set up in this work to verify the effectiveness and accuracy of the proposed imaging method. Results show that the defect imaging obtained by the multi-mode LW CTI method more accurately reflects the actual thickness variation, while neither S0 nor A0 single-mode method was able to distinguish thickness variation in the defect region. Theoretical and practical results prove that the proposed multi-mode LW CTI method can more accurately quantify and visualize variable-depth defects in metal plates.

## Figures and Tables

**Table 1:** Structure parameters of single A0-mode EMAT and single S0-mode EMAT.

Structure Parameters	A0 Mode EMAT	S0 Mode EMAT
Working frequency (kHz)	270	700
l (mm)	4.2	3.6
Outer diameter of the coil (mm)	37.8	32.4
Inner diameter of the coil (mm)	12.6	10.8
Number of windings	24	24
